# Supplementary figures and images for: “Tranq-dope” overdose and mortality: lethality induced by fentanyl and xylazine
Source: Front Pharmacol. 2023 Oct 26;14:1280289. doi: 10.3389/fphar.2023.1280289 (PMC10637371; doi:10.3389/fphar.2023.1280289)

**Supplemental Figure 3**


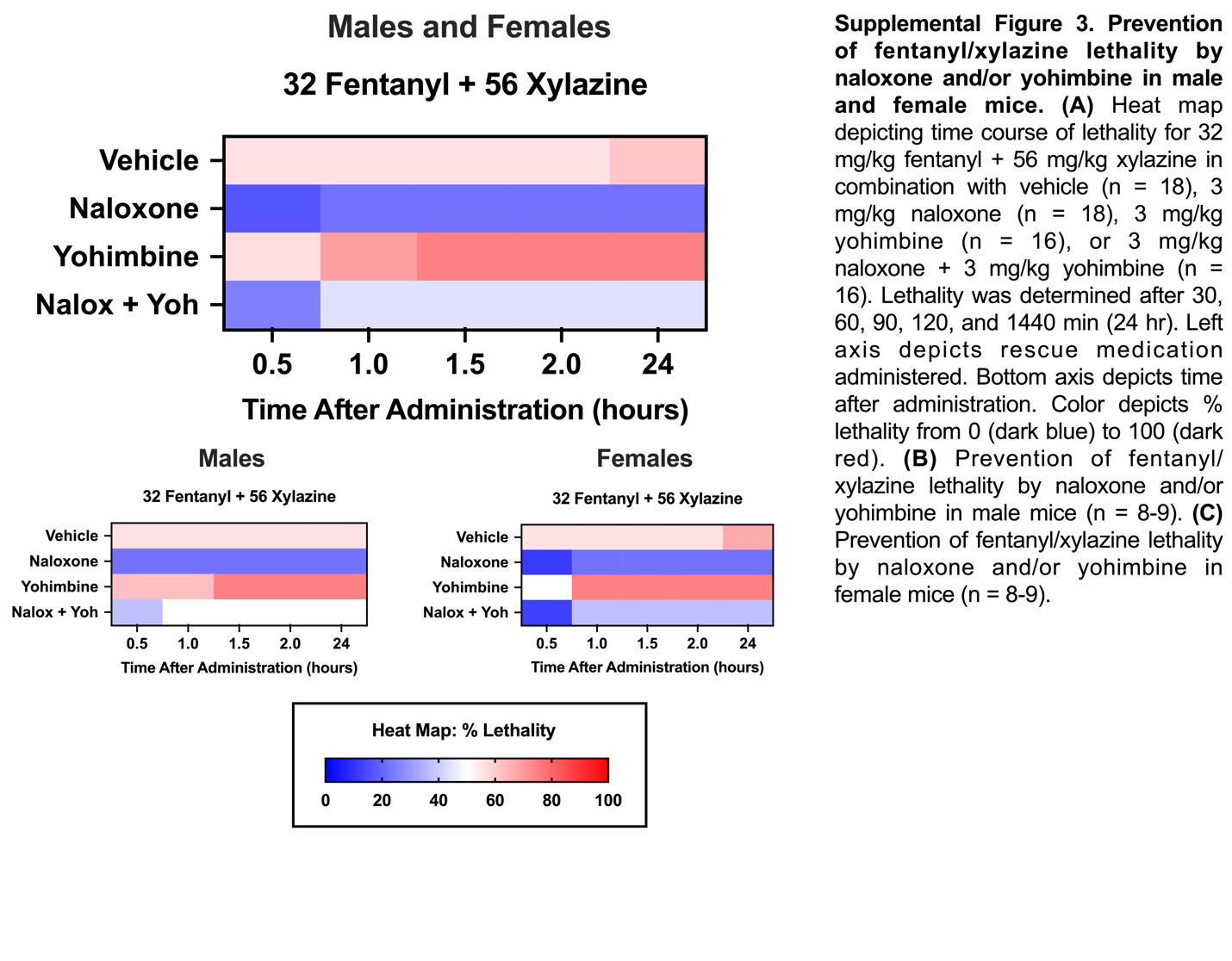

Supplement: Supplementary file 1 [file DataSheet3.DOCX]

**Supplemental Figure 2**


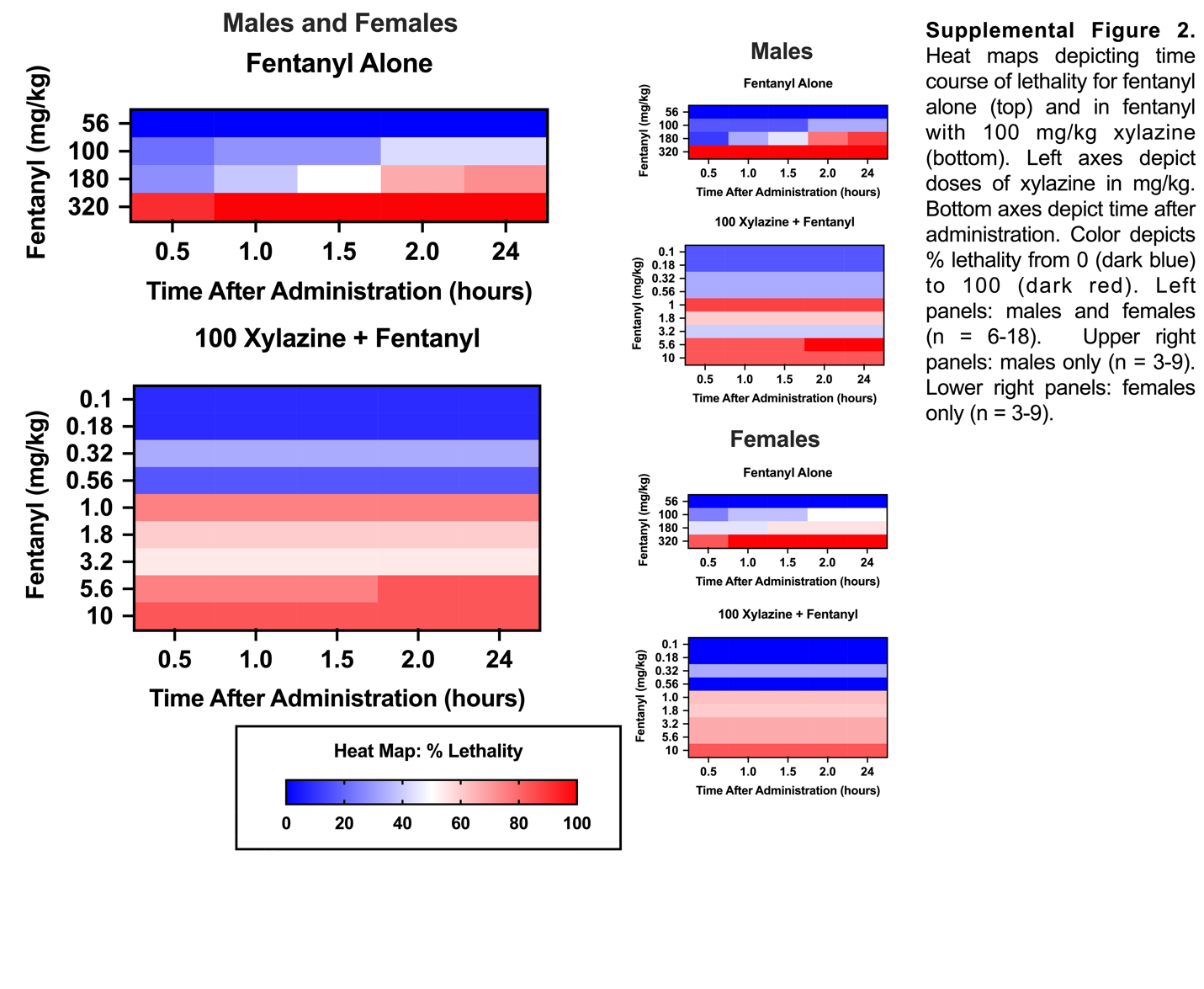

Supplement: Supplementary file 2 [file DataSheet2.DOCX]

**Supplemental Figure 1**


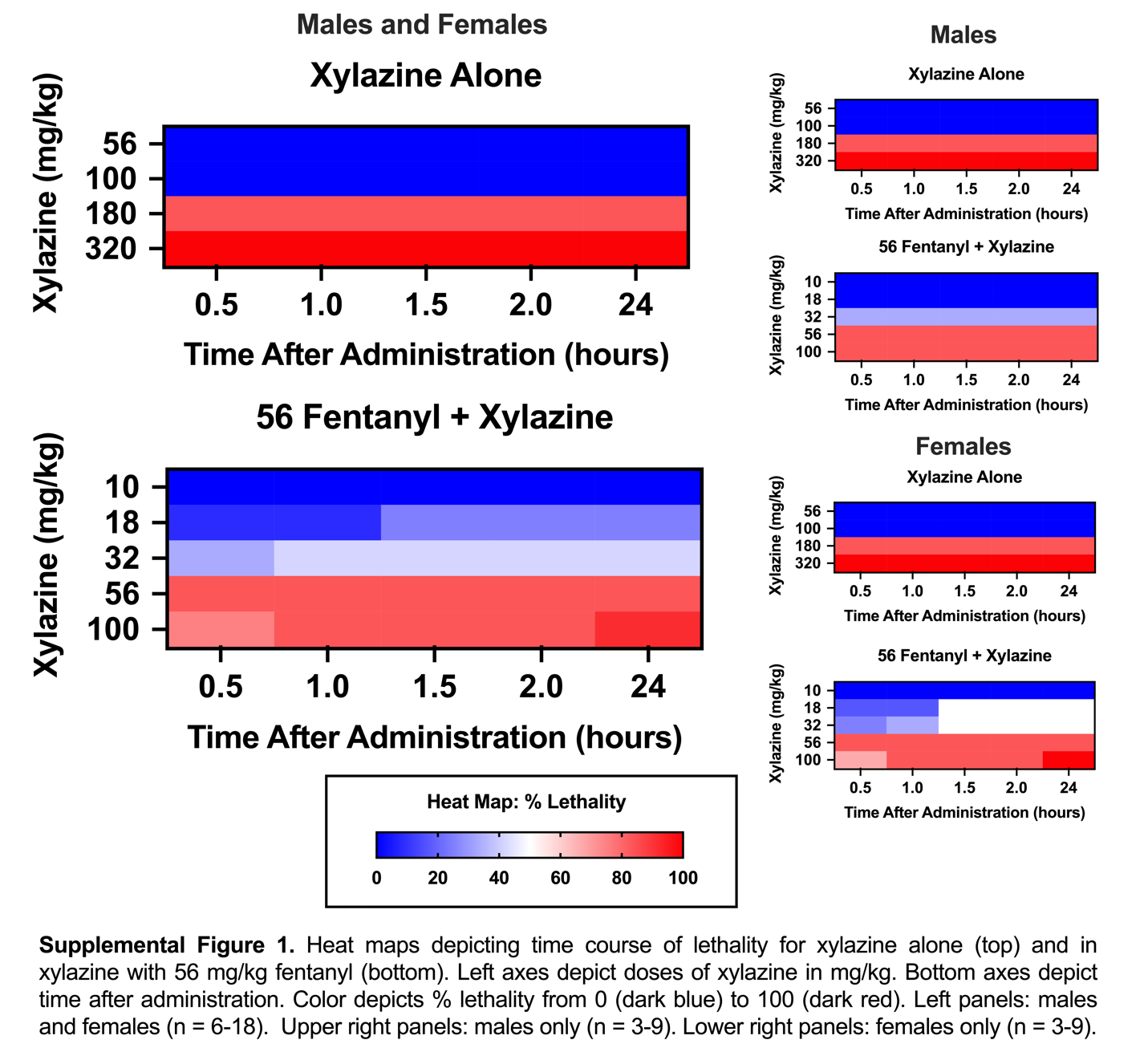

Supplement: Supplementary file 3 [file DataSheet1.DOCX]
